# Selectivity Enhancement by Using Double-Layer MOX-Based Gas Sensors Prepared by Flame Spray Pyrolysis (FSP)

**DOI:** 10.3390/s16091437

**Published:** 2016-09-06

**Authors:** Julia Rebholz, Katharina Grossmann, David Pham, Suman Pokhrel, Lutz Mädler, Udo Weimar, Nicolae Barsan

**Affiliations:** 1 Institute of Physical Chemistry, University of Tübingen, Auf der Morgenstelle 15, Tübingen 72076, Germany; Julia.Rebholz@ipc.uni-tuebingen.de (J.R.); katharina.grossmann@ipc.uni-tuebingen.de (K.G.); upw@ipc.uni-tuebingen.de (U.W.); 2 IWT Foundation Institute of Materials Science, University of Bremen, Badgasteiner Str. 3, Bremen 28359, Germany; dpham@iwt.uni-bremen.de (D.P.); spokhrel@iwt.uni-bremen.de (S.P.); lmaedler@iwt.uni-bremen.de (L.M.)

**Keywords:** logic device, selectivity, flame spray pyrolysis, gas sensor, double-layer sensor

## Abstract

Here we present a novel concept for the selective recognition of different target gases with a multilayer semiconducting metal oxide (SMOX)-based sensor device. Direct current (DC) electrical resistance measurements were performed during exposure to CO and ethanol as single gases and mixtures of highly porous metal oxide double- and single-layer sensors obtained by flame spray pyrolysis. The results show that the calculated resistance ratios of the single- and double-layer sensors are a good indicator for the presence of specific gases in the atmosphere, and can constitute some building blocks for the development of chemical logic devices. Due to the inherent lack of selectivity of SMOX-based gas sensors, such devices could be especially relevant for domestic applications.

## 1. Introduction

Recent years witnessed an explosion of the consumer microelectromechanical systems (MEMS) sensor market as the cell phone evolved into a powerful computer and the development of smart devices started. Consequently, for gas sensors based on Semiconducting Metal Oxides (SMOX), these developments open up new application fields such as indoor air quality monitoring in smart buildings or car ventilation, etc., as their use offers many advantages. These include high sensitivity to many toxic or explosive gases, simple measuring technique, the possibility for miniaturization and mass production, low cost, etc. [1,2]. One of their most significant limitations is the lack of selectivity, as the surface of the metal oxide reacts with many different gases, changing the measured electrical resistance. Interfering gases—even if they are only present in trace amounts—and variations in the background level of humidity can significantly change the sensor signal, which leads to uncertainty in the interpretation of sensor data. This is especially a problem in new application fields; for instance, when sensors are integrated into buildings. In a long-term vision, the sensor network should be able to identify the nature and origin of the response in order to act correctly. As an example, the cross-sensitivity between CO and ethanol is a challenge, because it should be clear if the sensor resistance changes due to the presence of a toxic gas or because somebody is just drinking a glass of wine. The previously applied countermeasure is the use of charcoal filters, which are included in the packaging of the sensor element [1]. The method is based on the fact that ethanol or other volatile organic compounds (VOCs) are far more reactive to surface-catalyzed decomposition than CO [2]. However, they suffer from saturation effects when the concentration of interfering gases or the exposure time is too high [1], and it will be very difficult to miniaturize them to the extent needed (e.g., for their integration in a smart phone). Other concepts are also applied to achieve selectivity [3]: Fine-tuning of the sensing material and operation conditions [4] and the use of sensor arrays [5,6] or specific filter materials (except charcoal) [7,8,9,10,11,12,13]. For the latter, gas detection with multilayer sensors consisting of a sensing layer and a filter layer on top was reported in previous studies. Generally, there are different concepts of how to increase the selectivity in this way:

(1) Physical filters hinder interfering gases to reach the sensing layer. For example, compact films of SiO_2_ on top of the sensing layer act as molecular sieve for selective H_2_ detection [8,13].
(2) Chemical filters can either eliminate the interfering gas by catalytic conversion into an inert product (for example, by depositing metallic films such as Pd, Pt, or Rh [10] or porous layers of metal oxides [8] on top of the sensing layer) or can influence the chemical reaction of the analyte in the sensing layer to enhance the desired response. In [7,14,15], an increase of sensitivity to a certain target gas was reported, which was achieved by the presence of a second noble metal-loaded Al_2_O_3_ layer.

Furthermore, recent studies showed that double-layer gas sensors using different combinations of metal oxides can have a positive effect on the gas response [15,16,17].

In the present study, we focus on a combination of functional layers of metal oxides that show different sensing properties in order to create a device which is able to distinguish between interfering and target gases; in this specific case, ethanol and CO. The investigations show that these multi-layers respond differently to individual target gases or even gas mixtures in comparison to the single layers. The use of the relationship between the resistances of single-layer and double-layer sensors (two single layers combined) opens up the possibility of gaining more information and improving the selectivity.

The functional layers are realized by direct thermophoretic deposition of nanoparticles synthesized by flame spray pyrolysis (FSP). FSP is a nanoparticle aerosol synthesis process that offers fast (within minutes) and dry (gas-phase) layer deposition. In comparison to conventional thick film SMOX sensor fabrication—where the deposition of pre-processed powders has to be done in an additional step after material synthesis—highly porous polycrystalline thick film layers can be realized by FSP in just one step [8]. Thus, FSP is a time efficient in situ technique for the direct deposition of the sensing material with the possibility of multi-layer fabrication [7,18,19,20,21]. Its flexibility allows for easy doping, and the appropriate choice of precursors and solvents results in a homogeneous distribution of dopants throughout the material. In the past, one drawback of the method was the high baseline resistance, which is related to the extremely high porosity of the layers. However, it was shown in recent studies [21] that by addition of antimony, the baseline resistance can be decreased by up to two orders of magnitude in dry air. Here, for illustration of the concept, the following sensing layers were chosen: 1 wt % Sb–0.01 wt % Pt-SnO_2_ alone or covered with 3 wt % Pd-SnO_2_ and 3 wt % Pd-SnO_2_ alone. Hence, a three sensor array was built and tested using two sensors based on individual single layers and one corresponding to the double layer (layer below//layer on top): 1 wt % Sb–0.01 wt % Pt-SnO_2_//3 wt % Pd-SnO_2_.

## 2. Materials and Methods

Sensor fabrication and material preparation: Tin 2-ethylhexhanoate (Strem Chemicals, Kehl, Germany, 99.5% pure), platinum acetylacetonate (Alfa Aesar, Ward Hill, MA, USA, 99.9% pure), antimony (III) propoxide (Sigma Aldrich, Munich, Germany, 99.9% pure), and palladium (II) acetylacetonate were used as metallo-organic precursors dissolved in a highly combustible organic solvent such as xylene to make the total metal concentration of 0.5 M. For the synthesis of pure SnO_2_, 50 mL of Tin (II) 2-ethylhexanoate precursor diluted (0.5 M by Sn) in xylene was used for spraying. For 3 wt % Pd-SnO_2_, 263 mg of palladium (II) acetylacetonate was dissolved in in 50 mL of 0.5 M Sn-precursor for spraying. Similarly, for 1 wt % Sb–0.01 wt % Pt-SnO_2_, 0.5 mL of Sb precursor was mixed with 50 mL of 0.5 M Sn-precursor. It should be noted here that the 0.01% of Pt promoter (0.6 mg Pt(acac)_2_ per 50 mL of the precursor–solvent combination) is added over 100% Sb-Sn combination. These precursor solutions were combusted using flame spray pyrolysis (FSP) to obtain ultrafine and highly crystalline particles. During combustion, the liquid precursor was supplied at the rate of 5 mL/min using a syringe pump. The precursor was then atomized by a two-phase nozzle with 5 L/min O_2_ at a constant pressure drop of 1.5 bar at the nozzle tip. The spray is ignited by a supporting CH_4_ and O_2_ premixed gases (1.5 L/min, 3.2 L/min) forming a self-sustaining spray flame. The particles are formed by reaction, nucleation, surface growth, coagulation, and coalescence in the spray flame environment and collected on glass fiber filters [22]. The sensing layer was directly in situ deposited onto comb-like structured pre-deposited Pt electrodes (30 µm electrode spacing) with the flame spray. The sensors were fixed on a copper support facing down towards the flame at a height of 25 cm from the nozzle, and the particles were thermophoretically deposited on the sensing area (2.5 mm × 5 mm) of the substrates [19]. For the fabrication of single-layer sensors (1 wt % Sb–0.01 wt % Pt-SnO_2_ and/or 3 wt % Pd-SnO_2_), the multiple sets of sensors were fabricated using each spray batch. However, for the double-layer sensor fabrication (1 wt % Sb–0.01 wt % Pt-SnO_2_//3 wt % Pd-SnO_2_), similar sensors were first coated with 1 wt % Sb–0.01 wt % Pt-SnO_2_ material followed by spraying 3 wt % Pd-SnO_2_ material on top of the previously coated sensors. X-ray diffraction and refinement of the patterns: The XRD-pattern of the freshly prepared nanoparticles (1 wt % Sb–0.01 wt % Pt-SnO_2_ and/or 3 wt % Pd-SnO_2_) were refined using Bremen Rietveld Analysis and Structure Suite (BRASS), followed by extracting the structural parameters. Background, scale factor, unit cell parameters, and Gaussian as well as Lorentzian peak width parameters were simultaneously refined, followed by crystallite size and microstrain analyses. For the pattern refinement, the structural model for SnO_2_ (ICSD-39173) with space group (P42/MNM) [a = b = 4.7384, c = 3.1881, α = β = γ = 90°] was used. Since the addition of Sb and/or Pt was ≤1%, the refinement was conducted using only the structural model of SnO_2_. The quality of Rietveld refinement was evaluated in terms of the usual R factor (R_wp_) and the background corrected residual R_p_ [23]. A volume-weighted average crystallite size (d_XRD_) and the root-mean-square lattice micro strain for each of the promoted and non-promoted SnO_2_-based sensing materials were determined from the line-broadening analysis. The instrumental contribution to the peak broadening was taken into account during the full profile fitting using instrumental parameters derived from a fit of standard crystalline LaB_6_.

Brunauer-Emmett-Teller (BET) measurements: BET measurements were carried out using a Quantachrome NOVA 4000e Autosorb gas sorption system. The powders were placed in a test cell and allowed to degas for 2 h at 200 °C in flowing nitrogen. The BET isotherm measurement using nitrogen as adsorbent at 77 K and relative pressure P/P_0_ over the range of 0.01–0.99 was considered. From the plot of [(P/P_0_)/w(1 − P/P_0_)] versus [P/P_0_] ranging between 0.05 and 0.3, straight lines were obtained, with the correlation coefficient being greater than 0.999. The BET surface area measurement is related to an average equivalent primary particle size, given by the equation d_BET_ = 6/(_p_·SA) [24], where d_BET_ is the average diameter of a monodisperse particle, S_A_ represents the measured surface area of the powder, and ρ is the theoretical density.

Transmission Microscopy (TEM) imaging: For TEM specimen preparation, a small portion of the sample (~1–2 mg) was dispersed in 5 mL of ethanol (AR grade, Strem) in an ultrasonic bath and sonicated for 15 min. A drop from an eye dropper of this dispersion sample was placed on a nickel grid coated with carbon film (Cu grid was not used, as it would interfere with the Cu in the sample during extraction of the quantitative elemental data). The samples were dried in ambient air, and large regions of the sample were scanned before the investigation of the particle morphology. The low- and high-resolution TEM of the sample and the corresponding selected area electron diffractions (SAED) were examined by transmission electron microscopy (TEM) on a FEI Titan 80/300 microscope equipped with a Cs corrector for the objective lens, a Fischione high angle annular dark field detector (HAADF), GATAN post-column imaging filter, and a cold field emission gun operated at an acceleration voltage of 300 kV.

## 3. Results

**Figure 1:**
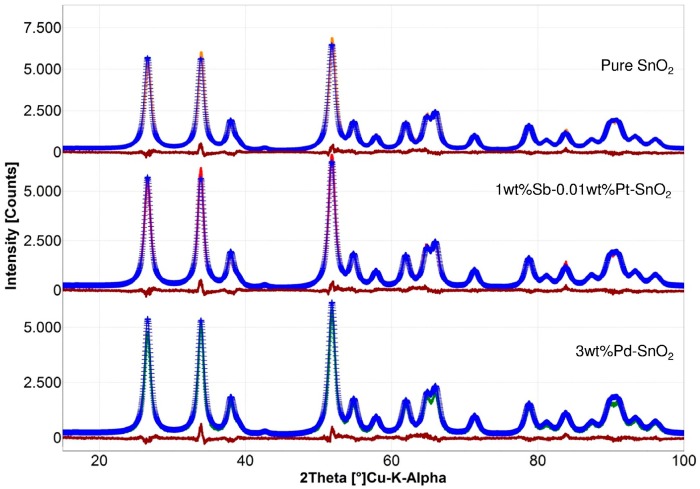
The Rietveld analysis of the XRD patterns of pure SnO_2_ (upper panel), 1 wt % Sb–0.01 wt % Pt-SnO_2_//3 wt % Pd-SnO_2_ (middle panel) and 3 wt % Pd-SnO_2_ (lower panel).

The characterizations of the dry particles were conducted to confirm that the particles obtained on the sensor substrates are identical to those collected as powders. The particles obtained from FSP were ultrafine and highly crystalline, as shown in Figure 1.

**Figure 2:**
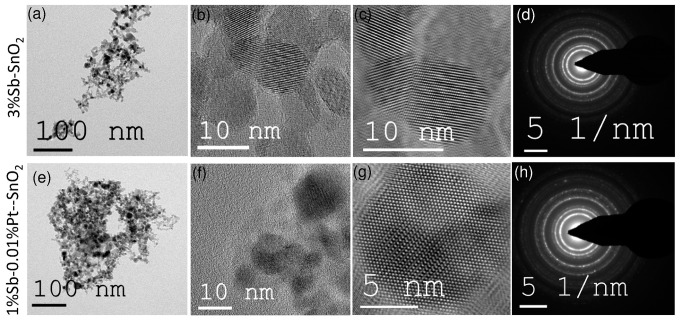
Low-resolution, high-resolution, Fourier-transformed image, and selected area diffraction pattern of 3 wt % Pd-SnO_2_ (**a**–**d**); and 1 wt % Sb–0.01 wt % Pt-SnO_2_ sensing materials (**e**–**h**).

The particles collected in the filter unit (very similar to those from the sensing substrates) were characterized using BET and XRD measurements. The specific surface areas of pure SnO_2_, 1 wt % Sb–0.01 wt % Pt-SnO_2_, and 3 wt % Pd-SnO_2_ nanoparticles were found to be 96.1, 93.4, and 100.3 m^2^/g, which are equivalent to 8.9, 9.2, and 8.6 nm, respectively. The crystallite sizes (d_XRD_) of 5.5, 5.6, and 5.7 nm for pure SnO_2_, 1 wt % Sb–0.01 wt % Pt-SnO_2_, and 3 wt % Pd-SnO_2_ were derived using Rietveld refinement of the XRD patterns. Although the crystallite sizes were slightly lower than the than the BET primary particle sizes (d_BET_) of different particles, both the techniques showed similar sizes for pristine and Sb and/or Pt supported particles. The low- and high-resolution TEM imaging showed highly crystalline particles (see Figure 2).

The particle sizes derived from TEM are approximately 10 nm (measured for at least 100 particles), which reasonably agree with the crystallite sizes obtained from Rietveld analysis. The well-formed ring patterns of the nanoparticle also support a highly-crystalline nature of the sensing materials. The sensors after in situ deposition with 1 wt % Sb–0.01 wt % Pt-SnO_2_ covered on the top with 3 wt % Pd-SnO_2_ were analyzed using focused ion beam (FIB) to determine the layer thickness of the first layer (1 wt % Sb–0.01 wt % Pt-SnO_2_), the second layer (3 wt % Pd-SnO_2_), and the total thickness of the combined sensing layer.

**Figure 3:**
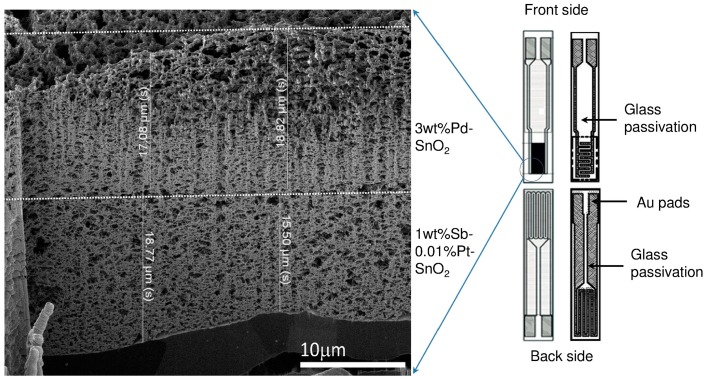
The focused ion beam (FIB) image of a double sensing layer composed of 1 wt % Sb–0.01 wt % Pt-SnO_2_//3 wt % Pd-SnO_2_. The total layer thickness was found to be approximately 35 (±2) μm (**Left**); Layout of the sensor substrates: Interdigitated platinum electrodes with trace/gap of 10 µm, 30 µm, and 100 µm, respectively, platinum heater on the backside. Heater and conductive pads to the electrodes are passivated (**Right**).

**Figure 4:**
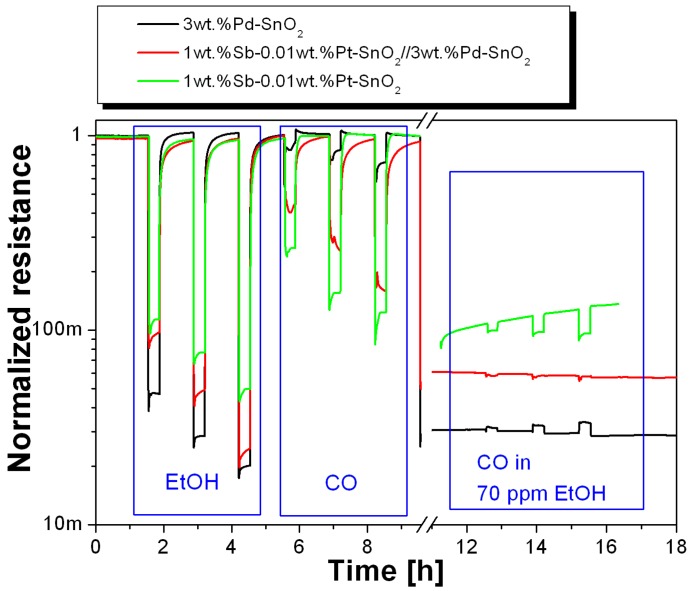
Sensing results at 300 °C in dry air for the system 1 wt % Sb–0.01 wt % Pt-SnO_2_//3 wt % Pd-SnO_2_.

The results showed that the upper layer covered with 3 wt % Pd-SnO_2_ and the lower layer (1 wt % Sb–0.01 wt % Pt-SnO_2_) had thicknesses of ~18 (±1) and 17 (±2) µm, respectively, while the total thickness of the sensing layer was found to be ~35 (±2) µm (see Figure 3, left panel). The thickness derived from FIB showed that the consistent layer thickness is possible using versatile flame spray pyrolysis. The substrates onto which the sensing layer was deposited are shown in Figure 3. The three sensors of the system were measured simultaneously at 300 °C operation temperature during exposure to 30 min pulses of ethanol (45, 90, 170 ppm) and CO (20, 50, 100 ppm) in dry air (see Figure 4). Afterwards, 20, 50, and 100 ppm CO was exposed in a background of 70 ppm ethanol. The Pt electrodes on top of the substrates were connected to an electrometer in order to measure the sensing layers’ resistance as a function of the ambient gas concentration.

## 4. Discussion

The results from the measurements performed as described in the experimental section are presented in Figure 4, where the time dependence of the normalized resistances is shown. The normalization factor is the initial baseline value of the sensor resistance. The single-layer sensor doped with Pt and Sb changes its resistance around one order of magnitude during exposure to ethanol, and is also sensitive to CO. Furthermore, there is a resistance decrease when the sensor is exposed to CO in a background of ethanol, which means that ethanol and CO have an additive effect.

The 3 wt % Pd-SnO_2_-doped sensor has very different sensing properties, as it shows the largest response to ethanol and nearly none to CO. In contrast to the 1 wt % Sb–0.01 wt % Pt-SnO_2_, it increases its resistance when measuring CO in a background of ethanol. It seems that due to the presence of CO, less ethanol reacts at the surface to decrease the resistance of the sensor. One reason could be the occupation of reaction partners—most probably oxygen ions—by CO to form carbonates sticking to the surface. Consequently, for the reaction with ethanol, a lower concentration of reaction partners is available to decrease the resistance of the sensor. No spectroscopic evidence for the formation of these carbonates is available for FSP-prepared layers, because it is not possible to record meaningful operando Diffuse Reflectance Infrared Fourier Transform (DRIFT) spectra with the small amount of sensing material deposited directly onto the substrates. Nevertheless, the fact that Pd dramatically modifies the CO surface reaction path and the formation of carbonate species on Pd-doped SnO_2_ thick film gas sensors obtained by classical wet chemistry preparation techniques was recently presented in [25]. The surface chemical reactions are obviously very different for the two single-layer materials. For the 1 wt % Sb–0.01 wt % Pt-SnO_2_, such processes do not dominate, as the CO is still able to release electrons into the conduction band of the semiconductor, and the resistance decreases.

When both materials are combined to form a double-layer sensor, the changes in resistance due to gas interaction are somewhere in between the resistances of the single-layer sensors. The presence of the second layer changes the responses to CO and ethanol of the 1 wt % Sb–0.01 wt % Pt-SnO_2_-based sensor; namely, it increases the response to ethanol and decreases the response to CO. This means that the presence of the 3 wt % Pd-SnO_2_ layer activates the layer of SnO_2_ doped with Pt and Sb when measuring ethanol. The effect cannot be explained by a classical filter function, in which ethanol is converted to CO_2_ and H_2_O in the filter layer and therefore a decreased number of ethanol molecules reach the sensing layer—this effect would decrease the sensor response to ethanol. Instead, a sensitization effect can be seen, which takes place at the interface between the two layers. The influence of Pd doping in FSP layers was studied in [26], where it was found that the dominating effect of Pd is related to the decrease of free charge carriers, while the effect on the surface chemistry is less strong. Therefore, the sensitization effect can most probably be explained by the electronic interaction of the two layers. The response of the double-layer sensor to CO can be explained by the classical filter function: CO is converted to CO_2_ in the filter layer, and thus a lower amount of CO is detected in the sensing layer. Therefore, the response of the double-layer sensor is lower than the 1 wt % Sb–0.01 wt % Pt-SnO_2_ sensor.

For the measurement of CO in a background of ethanol, the response of the double-layer sensor is very low. The combination of all three sensors in an array can be used for a more complex evaluation:

The fact that the double-layer sensor shows values of the resistance/sensor signals in between the two single-layers opens up the following new principle for a logic decision: as an example of the added value provided by the proposed array, a parameter linking the difference between the sensors was used; namely, the resistance ratio of the single-layer sensors and the double-layer sensor:

R(single-layer)/R(double-layer)(1)

**Figure 5:**
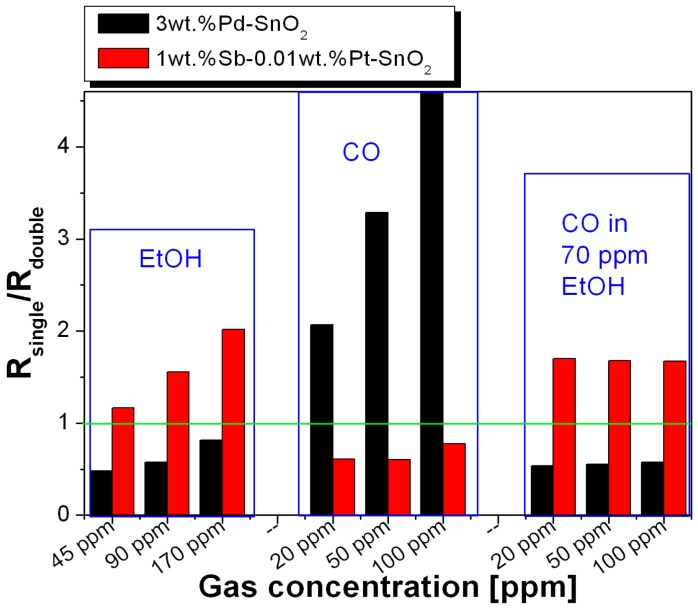
Resistance ratios of the single and the double layers.

The as-calculated values are presented in Figure 5 for the various test conditions.

The comparison of the gas composition behavior of the two ratios shown in Figure 5 demonstrates the capability of the proposed approach: all cases in which there is EtOH will be signaled by values of the ratio

R(1 wt % Sb–0.01 wt % Pt-SnO_2_)/R(double-layer) > 1 (2)

and values of the ratio

R(3 wt % Pd-SnO_2_)/R(double-layer) < 1 (3)

This fact is only possible because the double-layer sensor shows a mixed response of the two single layers that the array consists of. It clearly demonstrates the ability to distinguish whether ethanol is present or not. In the presence of CO only, the ratios show the opposite:

R(1 wt % Sb–0.01 wt % Pt-SnO_2_)/R(double-layer) < 1 (4)

and

R(3 wt % Pd-SnO_2_)/R(double-layer) > 1. (5)

On the basis of such an evaluation, a device can be developed which is able to identify the presence of a certain target gas in a gas mixture by combining the measured resistance ratio with a defined threshold value.

For subsequent quantification, it is necessary to use the calibration curves of the single- or double-layer sensors. Consequently, the results indicate that devices with combinations of different functional layers can be used to identify the presence of different target gases.

## 5. Conclusions

DC electrical resistance measurements on combinations of double- and single-layer sensors showed that the double-layers respond differently to exposure of CO and ethanol in comparison to the corresponding single-layer sensors, of which they consist. This fact was used to identify the presence of ethanol during CO measurement, which is especially important in domestic applications. It was realized by calculating resistance ratios of the single and double-layer sensors. The information from the comparison of the different resistance ratios with an appropriate threshold value—1 in the case of the system (1 wt % Sb–0.01 wt % Pt-SnO_2_ and/or 3 wt % Pd-SnO_2_)—can be used to make logic decisions about the presence of ethanol based on the different combinations of input signals. The findings could be the basis for more elaborated logic sensor devices using functional sensing layers. Therefore, additional material improvement and systematic studies of layer combinations will be the subject of future investigations in the direction of chemical logic sensor devices, in which logic gates could be directly implemented in the sensing layer’s nanostructure.
